# Ibogaine’s potential role in supporting reward system recovery across diagnostic boundaries

**DOI:** 10.3389/fphar.2025.1744383

**Published:** 2025-12-04

**Authors:** Mark Nicolas

**Affiliations:** Independent Researcher, Lampasas, TX, United States

**Keywords:** ibogaine, neuroplasticity, glial cell line-derived neurotrophic factor (GDNF), reward system, transdiagnostic psychiatry, addiction, post-traumatic stress disorder (PTSD), obsessive-compulsive disorder (OCD)

## Abstract

This article proposes that ibogaine’s induction of GDNF, modulation of glutamate and dopamine signaling, and reopening of plasticity represent a unified mechanism capable of restoring reward system fidelity across disorders. This includes addiction, obsessive-compulsive disorder (OCD), post-traumatic stress disorder (PTSD), and eating disorders, amongst others. These conditions are labelled as distinct and separate yet share disrupted dopaminergic and glutamatergic signaling within the mesocorticolimbic circuitry as a common underlying dysfunction, resulting in maladaptive reinforcement and motivational dysregulation. Ibogaine, a little-known indole alkaloid derived from *Tabernanthe iboga*, has shown distinct neurobiological effects that seem to restore normal reward processing through glial cell line-derived neurotrophic factor (GDNF) upregulation, receptor modulation, and enhanced neuroplasticity. Historical, preclinical, and observational evidence suggests that ibogaine’s mechanism of action is consistent with an action on underlying reward system dysfunction rather than only on surface symptoms. However, there are some safety concerns. Ibogaine causes hERG channel inhibition, so the therapy requires adherence to strict medical protocols. This review hypothesizes that ibogaine’s neurotrophic and receptor-modulating properties act as a unifying mechanism for restoring reward-system function across diagnostic boundaries. Unlike prior ibogaine reviews that focus on addiction, this paper integrates findings across trauma, compulsive behavior, and affective disorders through the common lens of reward-circuit dysfunction. The mechanisms described here support a theoretical framework that requires empirical validation, and the predictions derived from this model are outlined within the paper.

## Introduction

The treatment outcomes of most mental health disorders are not what people working in the field would hope for. Universities train future clinicians to prepare for burnout. Students of psychology are taught to expect high relapse rates and chronic treatment resistance. The repetitive attempts, high recidivism, and suboptimal outcomes that are common in current practice suggest a need for approaches that more directly address underlying mechanisms. Existing treatments such as extended courses of CBT often yield only modest benefits for a significant proportion of patients. Despite convergent evidence of reward circuit dysfunction across diagnoses, no unified mechanistic model has been proposed that links these conditions through a single pathway amenable to targeted intervention.

The majority of the field continues to treat psychiatric conditions as separate islands. Addiction, PTSD, eating disorders, and OCD each receive distinct diagnostic labels, care pathways, and pharmacological toolkits. However, those who have lived through or treated these disorders for a significant time recognize how easily they blur at the edges and that there seems to be a connecting thread underneath all the diagnosis criteria. The symptoms differ, but the internal landscape often seems the same, an imbalance of reward and regulation, reflecting reinforcement processes that have become dysregulated at the circuit level. Science is beginning to validate this lived experience.

New frameworks such as the Research Domain Criteria (RDoC) and the Hierarchical Taxonomy of Psychopathology (HiTOP) have sought to reorganize psychiatric conditions around underlying dimensions rather than surface symptoms. These models emphasize transdiagnostic constructs such as reward valuation, threat reactivity, cognitive control, and arousal regulation, many of which intersect directly with the mesocorticolimbic pathways discussed in this paper (Insel et al., 2010; Kotov et al., 2017). Computational psychiatry adds further precision by modeling disorders through disruptions in prediction error signaling and reinforcement learning algorithms, offering a mechanistic language for describing maladaptive valuation processes across conditions (Huys, Maia and Frank, 2016). The hypothesis advanced here aligns with these dimensional approaches, proposing that ibogaine’s actions may influence shared neural processes captured within these frameworks rather than symptoms specific to any single diagnostic label.

Building on these dimensional frameworks, it becomes necessary to examine the specific neural circuits that underlie valuation, motivation, and reinforcement learning. From a neurobiological perspective, addiction, trauma, and compulsive disorders share some overlap in the circuitry. The ventral tegmental area (VTA) sits atop the brain stem near the midbrain and communicates with the nucleus accumbens and prefrontal cortex through dopaminergic and glutamatergic pathways. Together, they form what is called the mesocorticolimbic reward system. This is the machinery that teaches the brain what is worth pursuing, what should be avoided, and what brings satisfaction. When this system is tuned properly, people orient toward meaningful reward and can sustain motivation. When it fails, behavior becomes distorted: individuals may engage in repetitive maladaptive behaviors, avoid previously rewarding activities, or become caught in self-reinforcing behavioral loops (Volkow and Morales, 2015; Volkow, Koob and McLellan, 2016).

The connecting thread between various mental health disorders reinforces the idea that the traditional diagnostic model is more symptom-based than biologically grounded. Instead of representing separate entities, these disorders could very well be variations of the same underlying dysfunction: a reward system that has become desensitized or otherwise dysregulated at the level of mesocorticolimbic circuitry. It is important to recognize that this convergence does not imply a single unified pathology. Contemporary models show that these disorders involve partially overlapping but distinct circuit disruptions, and the reward system represents only one common point of intersection rather than a totalizing explanation.

What has been lacking is an intervention capable of directly influencing these circuits in a way that restores more adaptive reward processing. Ibogaine’s pharmacology, including neurotrophic induction and receptor modulation within dopaminergic pathways, may support a degree of functional recalibration, positioning it as a potential transdiagnostic interventional tool for a range of disorders. This article advances the hypothesis that ibogaine’s modulation of dopaminergic, glutamatergic, and neurotrophic pathways enables recalibration of mesocorticolimbic circuitry, representing a shared mechanism relevant across addiction, PTSD, OCD, and eating disorders.

## Methodological approach

This narrative review synthesizes analysis of peer reviewed human and animal studies published between 1980 and 2024. Sources were identified through TAMUCT Library, PubMed, and PsycINFO using the search terms ibogaine, reward system, GDNF, dopaminergic regulation, and transdiagnostic. Studies were included if they contained mechanistic, neurochemical, or behavioral findings relevant to reward system modulation following ibogaine or noribogaine administration. This review is narrative in scope and does not constitute a systematic evidence synthesis. Studies were excluded if they focused solely on cardiovascular risk without neurobiological analysis or if they consisted only of anecdotal case descriptions without mechanistic or circuit level relevance.

## Pharmacological background and mechanistic overview

Ibogaine is an indole alkaloid extracted from the root bark of *Tabernanthe iboga*, a shrub native to the rainforests of Central West Africa. It induces a deep oneirogenic state that is physically demanding. During the acute phase individuals often report vivid visions and profound introspective or mystical experiences accompanied by temporary ataxia and nausea. For centuries, the Bwiti peoples of Gabon, Cameroon, and the Republic of the Congo have used iboga in initiation practice, personal growth, and healing. Within their initiative traditions, it is not regarded as a drug but as a teacher. To the Bwiti, iboga contains a plant spirit believed to reveal truth, confront suffering, and restore moral alignment within the community (Fernandez, 1982).

The symbolic framing of death and rebirth in these traditions can be viewed as broadly consistent with modern interpretations of transient windows of heightened neuroplasticity. Incorporating this anthropological context is useful because symbolic confrontation, narrative restructuring, and intensified autobiographical recall are known to modulate limbic salience networks and medial prefrontal integration. These processes may interact with ibogaine-induced plasticity windows, shaping how new emotional and cognitive associations consolidate during treatment.

Western researchers first isolated ibogaine in 1901, later marketing low dose extracts under the trade name Lambarène as a stimulant and antidepressant (Goutarel, Gollnhofer and Sillans, 1993). Ibogaine remained relatively obscure until the 1960s, when Howard Lotsof, an American researcher and former heroin user, observed that a single high dose eliminated withdrawal symptoms and reduced craving. This anecdotal finding initiated decades of underground practice and scientific inquiry (Alper, 2001).

Preclinical studies show that ibogaine operates differently from conventional detoxification agents. It modulates dopamine transporters, influences glutamatergic tone, and upregulates neurotrophic factors such as glial cell line derived neurotrophic factor (GDNF). This sequence of actions promotes dopaminergic neuron survival within the ventral tegmental area and nucleus accumbens (He and Ron, 2006; Carnicella et al., 2010; Marton et al., 2019; Iyer et al., 2021). By increasing GDNF, ibogaine and its metabolite noribogaine appear to support recovery of reward circuits disrupted by chronic stress and substance exposure.

The significance of GDNF upregulation extends beyond a biomarker shift. GDNF is a survival factor for dopaminergic neurons in the ventral tegmental area and substantia nigra. By engaging the GFRα1/RET receptor complex, it activates pathways that facilitate neuron survival, dendritic growth, and synaptic remodeling (He and Ron, 2006; Carnicella et al., 2010). These processes strengthen mesocorticolimbic circuitry and may help recalibrate how the brain encodes value and prediction.

Ibogaine’s effects on glutamatergic signaling complement these actions. Through mild NMDA receptor antagonism and stabilization of mGluR2/3 activity, ibogaine reduces excessive glutamate release associated with hyperexcitability and persistent maladaptive behavioral patterns (Glick, Maisonneuve and Szumlinski, 1998; Popik, Layer and Skolnick, 1995). This modulation supports extinction learning, allowing maladaptive reward and fear associations to be updated. Together, GDNF induction and glutamatergic modulation reopen a window of plasticity in which new emotional and behavioral patterns may be acquired, suggesting that therapeutic value depends on both pharmacological effects and post treatment learning processes (Marton et al., 2019).

Since ibogaine’s classification as a Schedule I substance in the United States in 1970, research has continued primarily in international settings. Observational studies in the Netherlands, Mexico, and New Zealand report rapid reductions in withdrawal and craving and higher abstinence rates than conventional detoxification, despite methodological limitations (Alper et al., 1999; Brown and Alper, 2018; Noller, Frampton and Yazar Klosinski, 2018). These findings parallel biological evidence showing that noribogaine persists for days and that GDNF upregulation can continue for weeks, indicating a potentially sustained neurobiological effect rather than a transient symptomatic change (Carnicella et al., 2010; Marton et al., 2019; Iyer et al., 2021).

Although this review focuses on dopaminergic, glutamatergic, and neurotrophic pathways because of their relevance to reward-system recalibration, ibogaine’s pharmacology is broader. Preclinical work shows that ibogaine and its metabolites interact with serotonergic receptors, monoamine transporters, opioid receptors, and other targets that likely contribute to both its therapeutic and adverse effect profile (Wells, Lopez and Tanaka, 1999; Iyer et al., 2021). For example, ibogaine inhibits dopamine and serotonin transport in rat brain synaptosomes and stabilizes transporters in noncanonical conformations, and iboga alkaloids have been reviewed as ligands with activity at multiple receptor systems that are implicated in affective and motivational regulation (Wells et al., 1999; Iyer et al., 2021; Marton et al., 2019). These findings highlight that ibogaine cannot be reduced to a single circuit level mechanism, and future work will need to clarify how these diverse receptor and transporter actions relate to the clinical phenomena described in observational studies.

Mechanistically, noribogaine shares several properties with ibogaine, including serotonin transporter inhibition and modest NMDA antagonism, but appears to exhibit reduced activity at certain targets such as kappa opioid and sigma receptors, although comparative quantitative data are limited (Popik, Layer, and Skolnick, 1995; Wells, Lopez, and Tanaka, 1999). A further clinical consideration is that ibogaine’s acute oneirogenic and autobiographical intensification phase may itself contribute to therapeutic change. Ethnographic and observational accounts consistently describe this phase as involving heightened autobiographical recall, confrontation of emotionally salient memories, and increased access to symbolic or narrative material (Fernandez, 1982; Alper, 2001). These features align with research showing that emotionally engaged autobiographical processing can modulate limbic salience networks and interact with periods of increased neuroplasticity (Pace and Heim, 2011; Paulus and Stein, 2010). Ibogaine induced neurotrophic signaling, particularly GDNF upregulation, creates a transient plasticity window (He and Ron, 2006; Carnicella et al., 2010), and it is plausible that the visionary state facilitates cognitive and emotional updating within this window. Because noribogaine does not reliably produce this immersive cognitive emotional state, it remains uncertain whether it would allow patients to utilize the same therapeutic learning processes that may contribute to outcomes in structured ibogaine treatment. Controlled comparisons will be required to determine whether noribogaine combined with magnesium can match the efficacy of ibogaine or whether the full phenomenological experience is needed to capitalize on the underlying neurobiological plasticity.

### Transdiagnostic evidence of reward-system dysregulation

Across addiction, PTSD, OCD, and eating disorders, biological evidence points to a common functional pattern: marked diminished sensitivity to natural rewards, excessive motivational significance for disorder-related cues, and maladaptive reinforcement learning (Volkow and Morales, 2015; Volkow et al., 2016; Frank, 2013; Lokshina, Nickelsen and Liberzon, 2021). In addiction, chronic substance use produces reduced dopaminergic responsivity in the ventral tegmental area and nucleus accumbens, directly impairing reward valuation and increasing the motivational salience of drug related cues (Volkow and Morales, 2015; Volkow, Koob and McLellan, 2016). In OCD, fronto striatal dysregulation alters dopaminergic and glutamatergic signaling in a way that reinforces compulsive action loops, reflecting a reward prediction and reinforcement impairment consistent with the proposed model (Denys et al., 2013; Dong et al., 2020). In PTSD, reduced activation in reward circuit nodes such as the ventral striatum contributes to anhedonia and threat biased reinforcement learning, aligning with the framework of disrupted reward system calibration (Nawijn et al., 2015; Lokshina, Nickelsen and Liberzon, 2021). In eating disorders, altered reward prediction error signals and atypical dopaminergic responses to food cues represent another manifestation of impaired valuation processes within mesocorticolimbic circuitry (Frank, 2013; Frank et al., 2018; Avena et al., 2012). Although direct clinical ibogaine data exist primarily for addiction, the reward-circuit features described above create a theoretical basis for cross-diagnostic relevance.

At the same time, it is important to acknowledge that mesocorticolimbic reward pathways do not operate in isolation. Disorders such as PTSD, OCD, and eating disorders also involve dysregulation in stress and interoceptive systems, including altered hypothalamic pituitary adrenal axis activity and heightened amygdala reactivity to threat (Pace and Heim, 2011). Habit circuitry within the dorsal striatum contributes to the shift from goal directed action to rigid stimulus driven responding that characterizes both compulsive behavior and substance use, linking these conditions to broader impairments in control over learned responses. Interoceptive regions such as the insula integrate bodily states with emotional and motivational signals and show altered activation across anxiety, mood, and addictive disorders (Paulus and Stein, 2010). Although ibogaine’s direct effects on these stress, interoceptive, and habit circuits remain under studied, the compound has documented actions across multiple receptor systems and monoaminergic pathways that extend beyond the dopaminergic and glutamatergic mechanisms emphasized here. These broader pharmacological effects make indirect modulation of these interconnected networks plausible and clinically relevant.

Taken together, this broader circuitry provides essential context for understanding why ibogaine’s dual action on dopaminergic and glutamatergic transmission, alongside GDNF induction, may intersect with the same processes implicated across these disorders. These combined effects may support more adaptive valuation and reinforcement processing, potentially allowing the brain to respond more appropriately to genuine rewards rather than maladaptive substitutes (He and Ron, 2006; Carnicella et al., 2010; Marton et al., 2019; Iyer et al., 2021).

### Clinical correlates across disorders: addiction, OCD, PTSD, and eating disorders

With the exception of addiction, no clinical ibogaine studies currently exist for the disorders discussed below. Each of these conditions contains substantial internal heterogeneity; for example, anorexia nervosa and binge-type eating disorders show different reward response patterns, and OCD subtypes vary in the degree and location of frontostriatal involvement, so any proposed mechanism must be flexible enough to accommodate these variations (Frank, 2013; Gillan et al., 2016). The following subsections interpret disorder-specific reward-process disturbances through a theoretical lens grounded in ibogaine’s known mechanistic actions.

### Addiction

Addiction is perhaps the clearest illustration of severe reward-system dysfunction. Nearly all addictive substances over-activate dopamine, forcing the brain to recalibrate by dulling its sensitivity to ordinary stimuli. This leaves individuals chasing stronger highs while deriving little pleasure from normal life (Volkow and Morales, 2015; Volkow et al., 2016). This pattern helps explain why substances produce strong reinforcing effects and why individuals may pursue escalating dopaminergic reward despite diminishing returns. In the end, the continued substance use alters the hedonic set point entirely. In open-label and observational studies, ibogaine appears to interrupt this process. Alper et al. (1999) reported that 25 of 33 patients experienced complete opioid withdrawal relief within 24 h of a single dose. Brown and Alper (2018) observed substantial reductions in withdrawal and craving with sustained abstinence in a subset of participants. Though these studies were not randomized, the reported outcomes suggest effect sizes and time courses that appear favorable when compared to many current therapies in addiction care.

#### Obsessive-compulsive disorder

OCD is generally framed as a disorder of anxiety and control. Reinforcement mechanisms also play a central role in the maintenance of these symptoms. Intrusive thoughts gain exaggerated motivational significance, and compulsions provide transient relief (reward) that perpetuates the cycle. In this context, fronto striatal dysregulation alters dopaminergic and glutamatergic signaling in a way that reinforces compulsive action loops, reflecting a reward prediction and reinforcement impairment consistent with the proposed model (Denys et al., 2013; Dong et al., 2020). Neuroimaging shows altered striatal dopamine signaling and fronto striatal coupling, showing patterns of dysregulation that overlap with those observed in addiction. Although no large scale ibogaine trials exist, its NMDA and kappa opioid receptor activity, combined with GDNF driven plasticity, make it plausible that ibogaine could influence the rigid behavioral patterns characteristic of this condition (Glick, Maisonneuve and Szumlinski, 1998; He and Ron, 2006).

#### Post-traumatic stress disorder

PTSD represents yet another distortion of reward learning. Chronic hyperarousal shifts neural processing toward threat avoidance rather than reward seeking. Functional imaging reveals decreased activity in the VTA and nucleus accumbens, patterns that are associated with anhedonia and emotional blunting (Nawijn et al., 2015; Lokshina et al., 2021; Torrisi, Leggio, Drago and Salomone, 2019). Ibogaine’s induction of GDNF and modulation of monoaminergic tone may reopen plasticity in these regions, potentially supporting new emotional learning rather than fear-based reactivity (Iyer et al., 2021).

#### Eating disorders

Eating disorders, again, reflect maladaptive reward processing. Restrictive and binge-purge behaviors become self-reinforcing as dopamine signaling adapts to the abnormal pattern. These behaviors can produce short-term reinforcement despite long-term negative consequences. Neuroimaging shows distorted reward prediction-error signals in anorexia and bulimia, consistent with dysfunctional valuation systems (Frank, 2013; Frank et al., 2018; Avena, Gold, Kroll and Gold, 2012).

Ibogaine’s broad receptor profile and ability to enhance GDNF expression suggest that it may have theoretical relevance for modulating these disrupted reward processes, warranting future exploration. It is important to note that anorexia nervosa often presents with blunted reward responsivity, whereas binge type eating disorders show heightened reward reactivity, a divergence demonstrated across neuroimaging work in these conditions (Kaye et al., 2009; Stice et al., 2008). A mechanism capable of moving valuation processes toward midline regardless of whether the deviation is hypo responsive or hyper responsive would be consistent with a transdiagnostic regulatory process rather than a disorder specific one.

### A synthesis supporting reward-system modulation and recovery processes

Collectively, the evidence suggests that these conditions may share partially convergent disruptions in reward valuation and reinforcement learning, rather than a single uniform root injury. The vulnerabilities involved appear overlapping but not identical, and the model proposed here is intended as a heuristic framework for those shared patterns rather than a claim of a singular causal lesion. Each of these disorders, whether the main symptomology is compulsion, avoidance, craving, or restriction, reflect a similar fundamental injury to the brain’s reward and valuation machinery.

Ibogaine does not only influence symptoms; it may support the recalibration of reward learning and emotional regulation processes. By reopening critical periods of plasticity through GDNF induction and recalibrating dopaminergic and glutamatergic tone, these mechanistic effects may influence how neural systems differentiate between threat and safety or process motivational signals. This alignment with shared reward-processing disturbances may help explain why ibogaine shows promise across divergent diagnoses; its mechanistic actions intersect with features common to these conditions. If supported by future research, these mechanisms may refine how we conceptualize and approach disorders that share disruptions in reward circuitry.

#### Testable predictions derived from the proposed model

If ibogaine’s therapeutic potential is driven by its capacity to recalibrate mesocorticolimbic reward circuitry through GDNF induction and modulation of dopaminergic and glutamatergic signaling, then several testable predictions follow. These predictions outline where the hypothesis can be supported, challenged, or refined through empirical work.

1. GDNF Expression Changes:

If the proposed mechanism is correct, individuals receiving ibogaine under controlled medical conditions would be expected to show measurable increases in GDNF expression in ventral tegmental area projections relative to baseline or an active placebo. Any such shifts should correlate with early behavioral changes in motivation or withdrawal-related symptoms.

2. Reward Circuit Responsivity:

Functional neuroimaging conducted before and after treatment would be expected to show enhanced engagement of the ventral striatum and other reward-related nodes during anticipation and reward processing. This pattern would be consistent with restored sensitivity to natural reward signals.

3. Reinforcement Learning Shifts:

Performance on reinforcement-learning tasks may demonstrate normalization of prediction-error signaling following treatment, reflected in reduced cue-driven responding and more adaptive reward-based decision making. These findings would support the role of ibogaine in influencing reinforcement processes.

4. Cross-Diagnostic Convergence:

If the mechanism is truly transdiagnostic, individuals with addiction, PTSD, OCD, or eating disorders may exhibit overlapping patterns of reward-circuit modulation despite differing clinical presentations. Convergent neural signatures across disorders would support the possibility of a shared underlying mechanism.

5. Distinctiveness Compared to Other Plasticity-Inducing Interventions:

Ibogaine may produce mechanistic signatures that differ from those observed with ketamine, classic psychedelics, or neuromodulation. In particular, ibogaine could generate a longer-lasting elevation of GDNF or more durable dopaminergic recalibration. Demonstrating such differences would help clarify whether ibogaine occupies a distinct position among neuroplasticity-inducing interventions.

Together, these predictions translate the central hypothesis into specific empirical targets. They outline a path through which the model can be refined, supported, or refuted, helping to define the next steps for mechanistic and clinical research.

### Counterarguments and limitations

Skeptics will note that much of the ibogaine literature relies on uncontrolled, self selected samples and lacks blinding or placebo conditions. Expectancy, ritualistic context, preparation, and integration practices may influence outcomes, and these interactions have not yet been systematically evaluated. Ibogaine’s polypharmacology complicates attribution, as its metabolite noribogaine contributes overlapping effects and additional alkaloids are present that remain insufficiently characterized. Furthermore, heterogeneity in treatment settings and follow up duration limits generalizability. These critiques show the clear need for more controlled clinical trials with standardized screening, dosing, and outcome measures. Until more structured studies are conducted, claims of efficacy must remain provisional regardless of how promising early outcomes appear.

Another important limitation involves neuroplasticity itself. Although ibogaine may promote a period of heightened plasticity, the direction of such changes is not inherently predetermined. Plasticity has the potential to reinforce either maladaptive or adaptive patterns depending on contextual factors. Without adequate preparation, therapeutic framing, or structured integration, there is a theoretical possibility that individuals could reinforce preexisting patterns if post-treatment environments or behaviors do not support adaptive learning. Neuroplastic change is only as beneficial as the conditions in which it unfolds. Ibogaine may increase the brain’s receptivity to new learning, but it cannot determine whether the learning that occurs is adaptive. This consideration underscores why education, integration, and environmental design are not ancillary to treatment but essential components of its safety and success (Lüscher and Malenka, 2011).

#### Safety, ethical use, and translational outlook

Ibogaine’s therapeutic potential must be weighed against known medical risks. When administered without appropriate safeguards, serious adverse events, including fatalities, have been documented. There are many medication contraindications, general ambulatory concerns due to ataxia, and a temporary but reliable cardiac impact. The compound can prolong cardiac repolarization (QT) by inhibiting the hERG potassium channel, increasing the likelihood of torsades de pointes in vulnerable individuals (Koenig et al., 2012; Meisner et al., 2016). Documented fatalities typically involve inadequate screening, polysubstance use, or electrolyte imbalance. Rigorous protocols that include cardiac evaluation, urinalysis, electrolyte monitoring, medication review, and continuous observation are essential. Typical exclusion criteria include QTc prolongation, structural heart disease, and concurrent QT-prolonging drugs. When safeguards are followed, the risk profile appears more manageable, although this remains an area requiring more rigorous systematic evaluation.

Genetic variability represents an additional and underrecognized layer of safety concern. Polymorphisms in metabolic enzymes can meaningfully alter both ibogaine and noribogaine exposure, shaping clinical effects and contributing to adverse outcomes. As noted by Boukandou and Aboughe (2023), variants in CYP2D6 can significantly shift the balance of biotransformation, either slowing metabolism and increasing cardiotoxic burden or accelerating conversion toward noribogaine and altering psychoactive intensity. Recent work has also clarified that transporters, rather than CYP3A4 metabolism, exert the primary influence over ibogaine disposition. Martins et al. (2022) demonstrated that ABCB1 and ABCG2 restrict ibogaine’s plasma levels and brain penetration, while human OATP1B1, OATP1B3, and CYP3A4 contribute little to overall pharmacokinetics. These findings suggest that CYP2D6 genotype and efflux transporter activity may be the most clinically relevant determinants of individual variability. Incorporating targeted genotyping for CYP2D6, and potentially ABCB1 or ABCG2, into pretreatment evaluation could therefore enhance safety, refine dosing decisions, and improve patient selection in future clinical protocols.

In addition to these safety considerations, emerging clinical work is beginning to clarify how ibogaine may function within medically structured environments. As the field looks ahead, future research should focus on controlled human trials that quantify ibogaine’s effects on specific neural circuits and neurotrophic markers. Preliminary findings from the Stanford MISTIC trial (Cherian et al., 2024) demonstrated that magnesium ibogaine therapy produced measurable clinical and neurophysiological improvements in veterans with traumatic brain injury. The study enrolled twenty individuals in an open-label design and served as the first modern, institutionally regulated trial of ibogaine in the United States. Its success contributed to subsequent public research investment in Texas totaling fifty million dollars. The results suggest that, when administered under strict medical oversight and combined with neuroprotective agents such as magnesium, ibogaine may engage neuroplastic processes in a manner that appears tolerable and safe. Integrating this approach with advanced neuroimaging and circuit-based models, like those used in neuromodulation and network-based psychiatry, could clarify ibogaine’s mechanisms and optimize dosing, safety, and therapeutic durability across diagnostic boundaries. Collectively, these findings support the rationale for continued institutional investigation of ibogaine as a potential modulator of reward-related neurocircuitry.

Going beyond pharmacology, ibogaine’s greatest promise may emerge when it is combined with comprehensive residential therapeutic communities. Facilities that focus on skill building, education, and behavioral reintegration while also providing synergistic therapies such as ART, CBT, CPT, EMDR, and MeRT could provide the structure necessary to translate neuroplastic change into lasting transformation. Ibogaine may increase receptivity to learning and self-regulation, but without proper up-front education and a return to environments that reinforce those capacities, there remains a possibility that prior behavioral patterns could re-emerge or worsen. It is plausible that embedding ibogaine treatment within a long-term residential framework focused on education, emotional literacy, and community accountability could support improved long-term outcomes among individuals with chronic addiction and trauma-related disorders. Such a model would pair neurobiological recovery with real-world rehabilitation, increasing the likelihood that neurobiological changes translate into durable behavioral improvements.

### Summary of evidence relevant to the hypothesis

Evidence across observational studies, neurochemical analyses, and clinical case data suggests that ibogaine may increase glial cell line derived neurotrophic factor expression, modulate dopaminergic activity in the ventral tegmental area, and influence glutamatergic signaling within reward pathways. Clinical outcome reports frequently describe reductions in withdrawal severity, decreased drug seeking behavior, and increases in perceived psychological clarity in the short term. In several documented cases, participants have described increased motivation, improved emotional regulation, or reduced compulsive drive following treatment. Although findings remain preliminary and heterogeneous, some alignment is observable across molecular, behavioral, and experiential levels.

It is important to clarify that the framework presented here is not intended to replace or redirect existing dimensional models such as RDoC, HiTOP, or computational psychiatry. Instead, ibogaine’s mechanistic profile actually reinforces and complements those frameworks by engaging several of the same domains they identify as cross diagnostic points of convergence. Its effects on valuation, prediction error, reinforcement learning, and circuit level modulation align with the constructs already central to these models. In this sense, ibogaine supports the ongoing shift toward mechanism based, circuit oriented explanations of psychopathology by offering a compound whose actions map directly onto the principles these frameworks articulate.

## Discussion

Psychiatry has long treated symptoms in parallel rather than addressing the shared mechanisms that generate them. The growing ability to identify convergent neural pathways allows for more unified models of addiction, trauma, and compulsive disorders. These conditions involve disruptions to reward valuation, motivation, and reinforcement learning, reflecting a pattern of functional impairment at the circuit level rather than isolated psychological dysfunction. Ibogaine may interact with these disruptions at the mechanistic level by influencing reinforcement processes, engaging neuroplastic pathways through glial cell line derived neurotrophic factor signaling, and modulating dopaminergic and glutamatergic activity. While the evidence remains preliminary and safety considerations require careful medical oversight, the emerging alignment among molecular, behavioral, and experiential observations is worth further investigation.

The mechanistic implications extend beyond reward circuitry. Processes supporting synaptic repair and network stabilization may also have implications for memory or motor pathways. If future research confirms these cross-system effects, ibogaine and related compounds could represent candidates for therapeutics that act not only on symptoms but potentially on underlying neural processes. This framework aligns with ongoing efforts in psychiatry to better understand shared neural mechanisms across diagnoses. Ibogaine may serve as one candidate intervention that can be examined within this broader movement toward mechanism-informed models.

The model advanced here yields several testable predictions. These include measurable changes in GDNF expression and reward circuit responsivity following ibogaine administration, normalization of reinforcement learning signals on computational tasks, and overlapping patterns of reward system change across diagnostic groups despite divergent symptoms. Future work will need to determine whether these predicted changes occur and how they relate to any observed clinical effects.
